# Endocytosis and the internalization of pathogenic organisms: focus on phosphoinositides

**DOI:** 10.12688/f1000research.22393.1

**Published:** 2020-05-15

**Authors:** Glenn F. W. Walpole, Sergio Grinstein

**Affiliations:** 1 Program in Cell Biology, Hospital for Sick Children, Toronto, ON, Canada; 2 Department of Biochemistry, University of Toronto, Toronto, ON, Canada; 3 Keenan Research Centre of the Li Ka Shing Knowledge Institute, St. Michael’s Hospital, Toronto, ON, Canada

**Keywords:** endocytosis, phagocytosis, macropinocytosis, phosphoinositides, inositides, signaling, traffic, pathogen

## Abstract

Despite their comparatively low abundance in biological membranes, phosphoinositides are key to the regulation of a diverse array of signaling pathways and direct membrane traffic. The role of phosphoinositides in the initiation and progression of endocytic pathways has been studied in considerable depth. Recent advances have revealed that distinct phosphoinositide species feature prominently in clathrin-dependent and -independent endocytosis as well as in phagocytosis and macropinocytosis. Moreover, a variety of intracellular and cell-associated pathogens have developed strategies to commandeer host cell phosphoinositide metabolism to gain entry and/or metabolic advantage, thereby promoting their survival and proliferation. Here, we briefly survey the current knowledge on the involvement of phosphoinositides in endocytosis, phagocytosis, and macropinocytosis and highlight several examples of molecular mimicry employed by pathogens to either “hitch a ride” on endocytic pathways endogenous to the host or create an entry path of their own.

## Introduction: phosphoinositides and the internalization of the extracellular milieu

**Figure 1.**
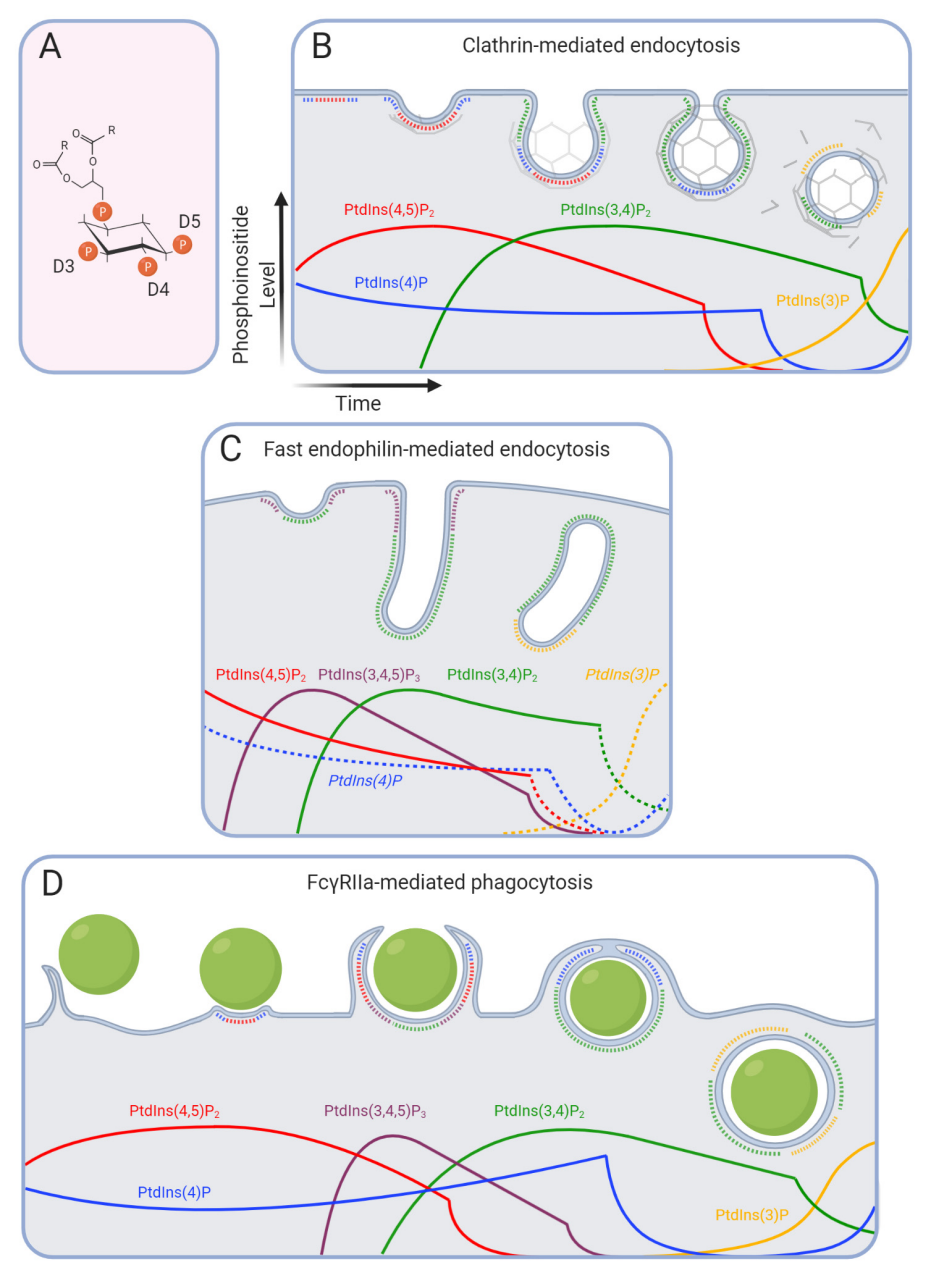
Phosphoinositide transitions during endocytic processes. ( **A**) The seven phosphoinositide species are derived from the same backbone through combinatory phosphorylation at positions D3, D4, and D5 of the inositol ring. Fatty acyl chains are abbreviated as (R) for simplicity. Phosphoinositides control and identify distinct stages of clathrin-mediated endocytosis ( **B**), fast endophilin-mediated endocytosis ( **C**), and Fcγ receptor-mediated phagocytosis ( **D**). Transitions that are speculative, i.e. not currently supported by experimental data, are labeled in italics and shown with dotted lines; they are predicted based on other endocytic pathways or on the presence of their precursor and/or the product of their hydrolysis. Although lipids intermix, phosphoinositides are drawn as single non-overlapping domains for simplicity. PtdIns, phosphatidylinositol.

The seven phosphoinositides, which form through combinatory phosphorylation of the inositol ring at positions D3, D4, and D5 ( Figure 1A), are present primarily on the cytosolic surface of biological membranes. By influencing the net charge of cellular endomembranes and directing the binding of ligands, phosphoinositides control the traffic and identity of organelles. Phosphoinositides are dynamic; their abundance and subcellular distribution are regulated in both time and space by active phosphorylation and dephosphorylation reactions as well as by transport between organelles via both vesicular and non-vesicular traffic. The resulting distinct—often inhomogeneous—accumulation of inositides can recruit proteins to specific organelles and even to subdomains therein. Such recruitment, which is often accompanied by allosteric activation, is made possible by specific protein domains that stereospecifically recognize defined phosphoinositide headgroups ^1– 3^.

In resting cells, phosphatidylinositol 4,5- *bis*phosphate—hereafter PtdIns(4,5)P _2_—predominates on the inner leaflet of the plasma membrane (PM), where it regulates ion transport and cytoskeleton anchorage and is a source of multiple second messengers ^2, 3^. PtdIns(4)P is abundant in the Golgi complex and is also found on the inner leaflet of the PM, where together with PtdIns(4,5)P _2_ it controls the non-vesicular counter-transport of phosphatidylserine to the PM ^4– 6^. The 3-phosphorylated species PtdIns(3,4,5)P _3_ and PtdIns(3,4)P _2_ are much less abundant in the PM of resting cells ^3, 7^. However, in response to a variety of cellular ligands including hormones, growth factors, and cytokines, the concentration of these rarer inositide species can be amplified drastically (by as much as 100-fold) to regulate key cellular processes. That dysregulated phosphoinositide metabolism underlies numerous human pathologies ^1– 3^ is a testament to the paramount importance of these lipids in cellular homeostasis.

Endocytosis is one of many key functions influenced by phosphoinositides. In virtually all cells, endocytosis is required for nutrient acquisition, cell-surface receptor internalization, and signaling regulation. In addition, some specialized cell types employ endocytosis for the surveillance and removal of foreign threats ^8– 12^. Strikingly, the endocytic machinery of the host cell can also be hijacked by some pathogens, which use molecular mimicry and deploy sophisticated toxins to gain entry to the intracellular environment ^13– 15^. Because they play critical roles in the formation and maturation of endosomes, phosphoinositides are often targeted by pathogens in their efforts to subvert host endocytic pathways.

Here, we review the initial events of a selection of endocytic pathways and their regulation by inositides, taking note of upstream regulators and downstream effectors. Finally, we highlight several examples of pathogenic organisms that have evolved ways to “hitch a ride” on endogenous endocytic pathways or have cleverly constructed uptake mechanisms by mimicking host pathways.

## Receptor-mediated endocytosis

### Clathrin-mediated endocytosis

In many cell types, clathrin-mediated endocytosis (CME) is the dominant endocytic pathway supporting housekeeping functions ^16^. The hallmark of CME and its distinction from other endocytic pathways is the formation of the clathrin triskelion lattice ( Figure 1B), which functions in concert with the large GTPase dynamin that mediates fission from the PM ^17^. Clathrin relies on the organized recruitment of over 50 adaptor and scaffolding proteins to form the clathrin-coated pit (CCP) ^8^. Initiation occurs at sites of low curvature enriched in PtdIns(4,5)P _2_ synthesized mainly by type I phosphatidylinositol 4-phosphate 5-kinases ^18– 22^. Adaptors such as the heterotetrameric AP-2 complex, CALM, FCHo1/2, and epsin bind this lipid at the PM, recruit clathrin, and bridge and cluster to cargo molecules ^23– 30^. Specific cargoes destined for endocytosis therefore become enriched at the PM with clathrin and PtdIns(4,5)P _2_.

Following cargo capture and clustering, its structural resistance must be overcome for the membrane to invaginate and form a spherical CCP ( Figure 1B). Such remodeling is elicited by cooperation between the clathrin lattice coat and scaffold proteins of the Bin, Amphiphysin and Rvs (BAR) domain family ^8^. BAR domains are dimeric membrane-binding modules that sense and induce membrane curvature/tubulation through their oligomerization ^31, 32^. Interestingly, BAR domains differ in their curvature and are recruited to CCPs in a sequential manner to promote neck constriction: F-BAR proteins with shallow curvature—such as FCH01/2—are recruited early, BAR domain proteins with intermediate curvature—like sorting nexin 9 (SNX9)—are recruited midway, and highly curved N-BAR proteins—such as endophilin and amphiphysin—are recruited at late stages ^23, 30, 32– 35^.

The hierarchical recruitment of BAR domain proteins is controlled through not only increasing membrane curvature but also changes in phosphoinositides. Maturation of the CCP requires the activity of the class II phosphatidylinositol 3-kinase (PI3K) C2α, which is activated at CCPs ^36, 37^ to locally generate PtdIns(3,4)P _2_ from PtdIns(4)P ^34^. PtdIns(3,4)P _2_ recruits the BAR domain-containing proteins SNX9 and SNX18 via their PX domains, which, together with AP-2, trigger their oligomerization and the constriction of the CCP neck ^38^ ( Figure 1B). Depletion of either PI3K-C2α or SNX9/18 leads to stalling of CCP necks in a U-shaped conformation ^34^. Actin polymerization also contributes to the shaping of the maturing CCP. The Arp2/3 complex stimulates branched F-actin assembly downstream of the nucleation-promoting factor (NPF) neural Wiskott-Aldrich syndrome protein (N-WASP) ^39, 40^. Both SNX9 ^41^ and FCHSD1/2, in the presence of PtdIns(4,5)P _2_/PtdIns(3,4)P _2_^42^, can activate N-WASP. Following constriction of the neck to an Ω-configuration, multiple BAR domain proteins recruit the fission executioner, dynamin ^31^.

Throughout maturation and fission, the levels of PtdIns(4,5)P _2_ appear to be controlled by 5-phosphatases of the synaptojanin (Synj) family. The p170 isoform of Synj-1 is recruited early during maturation ^43^, while the p145 isoform is recruited shortly before dynamin via the N-BAR protein endophilin ^33, 43– 45^. Interestingly, the dephosphorylation of PtdIns(4,5)P _2_ occurs preferentially in highly curved membranes; thus, Synj may aid in dynamin-mediated fission of the CCP neck ^46^. The clearance of PtdIns(4,5)P _2_ following scission is also necessary for vesicle uncoating ^47, 48^ and is supported in non-neuronal cells by other 5-phosphatases, including OCRL ^49^. Its dephosphorylated product, PtdIns(4)P, may directly support clathrin coat disassembly by recruiting auxilin2 and the ATPase HSC70 ^50, 51^. Following fission, PtdIns(4)P is hydrolyzed by the Sac2 phosphatase ^52, 53^, while remaining PtdIns(3,4)P _2_ persists until its hydrolysis by the early endosome-localized INPP4A/B phosphatase ^54, 55^. On early endosomes, PtdIns(3)P is synthesized mainly by Vps34, a class III PI3K, but contributions from class II PI3Ks have been noted ^56, 57^. PtdIns(3)P is also posited to support clathrin coat disassembly by recruiting auxilin1 and HSC70 ^50, 51^.

## Clathrin-independent endocytosis

Here we discuss fast endophilin-mediated endocytosis (FEME), a form of clathrin-independent endocytosis (CIE). We refer the reader to several recent reviews on other important CIE pathways which occur in various tissue types ^9, 58– 60^. These include clathrin-independent carriers/glycosylphosphatidylinositol-anchored protein-enriched endocytic compartments (or CLIC/GEEC), ultrafast endocytosis, generalized interleukin-2 receptor endocytosis, and caveolae.

### Fast endophilin-mediated endocytosis

Occurring predominantly at the leading edge of cells, FEME is an actin- and dynamin-dependent pathway that mediates the ligand-triggered uptake of several families of surface receptors ^61^. This includes G-protein-coupled receptors (α2a, β1 but not β2-adrenergic, dopaminergic D3 and D4, muscarinic acetylcholine receptor 4), receptor tyrosine kinases ([RTKs] EGFR, HGFR, VEGF, and PDGF among others), tyrosine receptor kinase B, and the interleukin-2 receptor (2Rα, 2Rβ, and γ _c_) in lymphocytes ^61– 65^. The N-BAR protein endophilin acts as a critical node in FEME, utilizing both its BAR domain as a scaffold for oligomerization on membranes and its numerous SH3 domain interactions to coordinate the capture of activated cargo with membrane bending and fission ^9^. To capture cargo, endophilin binds directly to proline-rich motifs present in cytosolic loops of many cargoes ^61^ or relies on intermediate adaptors in the case of RTKs ^62, 63^ and the tyrosine receptor kinase B ^64^.

Prior to receptor activation, endophilin forms prominent assemblies at lamellipodia through interaction with the protein lamellipodin and PtdIns(3,4)P _2_ ( Figure 1C) ^66^. The Pleckstrin Homology (PH) domain of lamellipodin associates with PtdIns(3,4)P _2_-rich regions of the membrane, and endophilin, in turn, is scaffolded onto lamellipodin through at least 10 binding sites ^66, 67^. In contrast to CME, PtdIns(3,4)P _2_ is generated at lamellipodia and during FEME through the production of PtdIns(3,4,5)P _3_ by class I PI3Ks ^68^ and its subsequent dephosphorylation by the phosphatases SHIP1/2 ^69^. Consistent with this phosphoinositide transition, RNA interference or pharmacological inhibition of SHIP1/2 decreases endophilin assembly and FEME, while PTEN reduction, which hydrolyzes position D3 of PtdIns(3,4,5)P _3_ and PtdIns(3,4)P _2_^70, 71^, increases endophilin assembly ^61^. PtdIns(4,5)P _2_ levels are controlled during FEME by the 5-phosphatase Synj, which is recruited to lamellipodia via endophilin ^47, 72^.

FEME promotes the fission of long tubular endosomes containing activated receptors. Indeed, endophilin can mediate extensive tubulation and vesicle formation when it attains high local concentrations ^44, 73^. However, a recent screen identified the F-BAR proteins FBP17 and CIP4 as being necessary for FEME initiation through the recruitment of SHIP2 and synthesis of PtdIns(3,4)P _2_^74, 75^. Interestingly, Cdc42 (a Rho-family GTPase) can recruit FB17 and CIP4 to the membrane when GTP bound, but GDP-bound Cdc42 (which is formed by the GAP activity of N-BAR proteins SH3BP1 and RICH1) terminates this cycle. After fission, PtdIns(3,4)P _2_ is cleared from tubular carriers by INPP4A/4B, releasing machinery for subsequent FEME cycles ^61^. In such a way, Cdc42 cycling together with inositides control the sequential recruitment of BAR and SH3 domain proteins for constriction and ultimately fission ^75^.

## Phagocytosis and macropinocytosis

### Phagocytosis

Innate immunity relies on phagocytosis to recognize, internalize, and inactivate potential pathogens such as fungi and bacteria. Phagocytosis is a receptor-mediated, actin-dependent endocytic pathway that internalizes cargo larger than 0.5 μm into a membrane-bound compartment termed the phagosome. The nascent phagosomal membrane, initially derived from the PM, is transformed (matures) through a highly regulated cascade of fusion and fission events to create an acidic, lytic luminal environment that is hostile to pathogens. Professional phagocytes of the myeloid lineage not only kill invading microorganisms but also present antigens generated upon their digestion to lymphocytes, coupling the innate and adaptive immune responses. Phagocytes also maintain tissue homeostasis by clearing endogenous debris and apoptotic cells. Such functional diversity necessitates an arsenal of phagocytic receptors capable of recognizing pathogen- or danger-associated determinants; these can be intrinsic to the target or the result of deposition of serum factors termed opsonins (e.g. immunoglobulin G [IgG] and complement component iC3b) ^10, 12^. Here we discuss primarily phagocytosis mediated by Fcγ receptors (FcγR) that recognize IgG. Not only is this type of phagocytosis the best studied to date but it also has the additional advantage that it can be reconstituted in non-phagocytic cells (fibroblasts, epithelial cells) by heterologous expression of myeloid FcγR ^76– 78^.

The cytosolic domain of FcγRs encodes immunoreceptor tyrosine-based activation motifs (ITAMs), which are substrates of tyrosine phosphorylation by Src-family kinases and Syk. The simultaneous engagement of multiple FcγRs by IgG coating the target particles triggers lateral receptor clustering and exclusion of cytosolic tyrosine phosphatases, steps that are absolutely necessary for the sustained signaling required for engulfment ^10, 12, 79, 80^. In the initial stages, PtdIns(4,5)P _2_ is present and modestly enriched at the site of particle engagement and in the actin-rich membrane pseudopods that zipper around the phagocytic target ( Figure 1D) ^81^. Multiple PIP5K isoforms that synthesize PtdIns(4,5)P _2_ from PtdIns(4)P localize to the phagocytic cup; their genetic perturbation has severe effects on particle engagement and uptake, altering actin remodeling in the nascent cup ^81– 83^. The Arp2/3 complex, activated by the NPFs WASP, N-WASP, and presumably also WASP-family verprolin homologous protein (WAVE) complexes, mediate the initial burst of actin polymerization associated with the extension of phagocytic pseudopodia. Recruitment of the NPFs occurs in response to Cdc42 and Rac stimulation via multiple adaptors, including Nck ^84^ and Grb2/Gab2 ^85^, downstream of activated ITAMs. PtdIns(4,5)P _2_ coordinates the activation of these NPFs in the extending pseudopods ^86– 91^.

While abundant in the pseudopods, PtdIns(4,5)P _2_ is rapidly cleared from the base of the phagocytic cup, becoming undetectable in the nascent phagosome ( Figure 1D). The local loss of the inositide marks a critical transition for particle internalization, as it demarcates the regional disassembly of F-actin that is seemingly essential for phagosome closure ^81, 92^. Indeed, artificially elevating PtdIns(4,5)P _2_ at the cup by overexpression of PIP5Ks sustains F-actin at the base of the cup and precludes particle uptake ^92^. The clearance of PtdIns(4,5)P _2_ from the nascent cup occurs via multiple pathways: phospholipase C (PLC)-mediated hydrolysis is thought to be the predominant mechanism ^81^, but PI3K-mediated phosphorylation to PtdIns(3,4,5)P _3_^93, 94^ (see below), focal exocytic insertion of endomembranes devoid of the inositide at the base of the cup ^95, 96^, and dephosphorylation by the phosphatases OCRL and INPP5B ^97^ also contribute. As a result of such phosphatase activity, the concentration of PtdIns(4)P spikes in the membrane of the nascent phagosome ( Figure 1D). PtdIns(4)P then declines abruptly after phagosome sealing, an effect attributed mainly to Sac2, although PLC may be partly responsible ^98^.

The 3-phosphorylated inositides also feature prominently in phagocytosis. PtdIns(3,4,5)P _3_ and PtdIns(3,4)P _2_ accumulate robustly in pseudopods and in the forming phagosomal cup ^94, 99, 100^ ( Figure 1D). The p85 regulatory subunit of class I PI3K can be recruited directly by activated ITAMs ^101^ or by other adaptor proteins ^85, 102, 103^ to mediate the synthesis of PtdIns(3,4,5)P _3_. The 5-phosphatases SHIP1 and SHIP2 are recruited and activated by both ITAMs and immunoreceptor tyrosine-based inhibitory motif domains (ITIMs) found in FcγRIIB and mediate the dephosphorylation of PtdIns(3,4,5)P _3_ to PtdIns(3,4)P _2_^100, 104– 108^. Analysis of the dependence of phagocytosis on PI3K signaling has revealed a peculiar disparity in the literature: although actin polymerization persists at sites of phagocytosis despite pharmacological inhibition of PI3Ks, the uptake of large (≥5 μm) but not small particles is inhibited ^99, 109– 111^. Several explanations for the phenomenon have been offered, the most compelling being that PI3K products signal the termination of Rho-GTPase signaling that is required for progression of actin polymerization around large targets. Following pseudopod extension, GTP hydrolysis by Rac and Cdc42 GTPases is necessary for termination of F-actin assembly at the base of the phagocytic cup, an event that is critical for the engulfment of large ^110, 112^ but not small particles ^92^. Accordingly, a recent study identified the RhoGAPs ARHGAP12, ARHGAP25, and SH3BP1 as being recruited to the phagocytic cup in a PI3K-dependent manner and established that they are required for large, but not small, particle internalization ^110^. Consistent with this interpretation, the overexpression of SHIP1—which is predicted to reduce PtdIns(3,4,5)P _3_—inhibits FcγR-mediated phagocytosis of large particles while its 5-phosphatase-dead counterpart (that exerts a dominant-negative inhibitory effect) or knockout of SHIP1 enhances phagocytosis of large particles ^106, 113^. Thus, products of class I PI3K activation signal the de-activation of Rho-family GTPases and actin disassembly ^110, 112^. Why is the conversion of phosphoinositides paramount to phagosome sealing? The continued *de novo* polymerization of actin along extending pseudopods is likely to exhaust one or more cytoskeletal factors. The clearance of PtdIns(4,5)P _2_ and synthesis of PtdIns(3,4,5)P _3_ likely orchestrate both the termination of actin polymerization and the disassembly of existing actin filaments at the base of the cup, which likely facilitate the recycling of limiting machinery components to pseudopods ^12, 100, 110^.

During phagocytosis, actin polymerization does not only occur at advancing pseudopods. Arp2/3 also induces the assembly of actin in discrete podosome-like structures that exert perpendicular pressure on the PM, promoting receptor engagement and zippering around the target ^114^. Podosome initiation in the nascent phagosome requires class I PI3K activity while their eventual disassembly depends on PtdIns(4,5)P _2_ hydrolysis.

To accommodate the protruding actin network and to envelop the targets, the PM needs to expand; this occurs by concomitant delivery and fusion of endomembranes to the phagocytic cup ^95, 115– 119^. The disruption of such focal exocytosis hampers pseudopod extension and impairs engulfment, especially that of large particles. Interestingly, this exocytic pathway is also dependent on PI3K activity ^96, 109^, possibly accounting in part for the preferential inhibition of large particle uptake by PI3K inhibitors. Although not yet demonstrated experimentally, by removing a physical barrier, the clearance of F-actin at the base of the cup may facilitate the fusion of exocytic vesicles; alternatively, PI3K products may directly stimulate the exocytic machinery.

PtdIns(3,4,5)P _3_ and PtdIns(3,4)P _2_ disappear from nascent phagosomes after a few minutes. PtdIns(3,4,5)P _3_ is converted to PtdIns(3,4)P _2_ by SHIP1/2 and the latter subsequently to PtdIns(3)P by INPP4A following closure of the phagosome ^100, 108, 120, 121^. Throughout closure and fission, phosphoinositides are likely to recruit and maintain membrane curvature-stabilizing/tubulating proteins of the BAR family such as amphiphysin ^122^, OPHN1, SH3BP1 ^110^, FBP17 ^123^, and SNX9 ^124^. In contrast to other endocytic pathways, the role of BAR proteins in promoting scission of the phagosome from the PM is not known. Finally, PtdIns(3)P is acquired by the phagosomal membrane soon after sealing and is obligatory for maturation to the phagolysosome stage ( Figure 1D). PtdIns(3)P acquisition is due in part to fusion with early endosomes, but *de novo* synthesis of PtdIns(3)P occurs via the PI3K Vps34 on the early phagosomal membrane ^120, 125^.

### Macropinocytosis

Evolutionarily conserved from protozoans to metazoans, macropinocytosis is an actin-based process utilized by innate immune cells to internalize bulk extracellular milieu, as well as membrane-bound structures, to survey for antigens and microbial components ^11, 126, 127^. It is also activated in cancer cells to drive elevated nutrient acquisition and support growth ^128^. Macropinocytosis is intimately dependent on membrane ruffling, driven by expansion of cortical actin networks underlying the PM. Membrane sheets must extend, curve, fuse at their margins, and ultimately undergo fission from the PM to enclose a large (>0.2–5 μm) macropinocytic vacuole ^129^; as such, not all ruffling leads to macropinocytosis ^130^. While dendritic cells and macrophages perform constitutive macropinocytosis ^127, 131^, here we focus on macropinocytosis induced in response to growth factors, chemokines, and Toll-like receptor agonists.

Much of the actin rearrangement in macropinocytosis revolves around PtdIns(4,5)P _2_ and signaling patches of PtdIns(3,4,5)P _3_/PtdIns(3,4)P _2_, which we discuss sequentially. PtdIns(4,5)P _2_ at the macropinocytic cup undergoes biphasic changes: increasing during the extension of F-actin-rich membrane sheets but then decreasing during sealing and internalization of the vacuole ^132^. The mechanism of the initial rise in PtdIns(4,5)P _2_ is unknown but is likely a consequence of activation of PIP5K isoforms, as described in other settings ^133^. Accordingly, PIP5K activators ^134^ such as phosphatidic acid, Rac1, and Arf6 are present and activated at macropinocytic cups ^135– 137^, and the activation of Rac1 can stimulate local PtdIns(4,5)P _2_ synthesis in ruffles ^138^. The elevation in PtdIns(4,5)P _2_ is consistent with the observed initial burst of F-actin at the base of the macropinocytic cup ^132^. The inositide could favor net actin polymerization by inhibiting barbed-end capping and/or by severing actin networks ^139^. PtdIns(4,5)P _2_-binding proteins such as profilin, cofilin, gelsolin, or capping protein could potentially mediate these effects. Additionally, PtdIns(4,5)P _2_ can activate the NPFs WASP and N-WASP to promote Arp2/3 activity ^140, 141^. At least four mechanisms are likely to contribute to the subsequent local decrease in PtdIns(4,5)P _2_ that accompanies macropinosome closure and fission: 1) decreased synthesis by inactivation or membrane detachment of PIP5K; 2) PLC-mediated hydrolysis that generates diacylglycerol and Ins(1,4,5)P _3_; 3) phosphorylation to PtdIns(3,4,5)P _3_ via class I PI3Ks (see below); and 4) dilution of the inositide upon focal exocytosis of endomembranes devoid of PtdIns(4,5)P _2_. Hydrolysis by 5-phosphatases is also conceivable ^142^.

Following ligand binding, G-protein-coupled receptors and RTKs together with Ras GTPases recruit class I PI3Ks to the PM ^143^, generating patches of PtdIns(3,4,5)P _3_ where macropinocytic cups form ^132, 144– 146^. The means whereby the localization of PtdIns(3,4,5)P _3_ is spatially restricted is not clear; cytoskeletal structures could confine its diffusion, but differential distribution of kinases and phosphatases could generate a standing gradient of diffusible phosphoinositide. Although PI3K activity is not required for ruffling, both genetic and pharmacological approaches point to an essential role of class I PI3Ks in completing macropinosome closure ^99, 147– 149^. Modulation of small GTPase activity is likely to mediate the effects of PtdIns(3,4,5)P _3_; a variety of GAPs and GEFs specific to GTPases of the Arf and Rho families are regulated by the inositide ^150– 152^. By activating Rac1 and its effector Pak1 ^153^, PtdIns(3,4,5)P _3_ coordinates the formation of rings of the SCAR/WAVE complex that promote the extension of branched actin along the macropinocytic cup walls by stimulating Arp2/3 ^146, 154^. Indeed, the Arp2/3 complex delineates the border of forming macropinocytic cups ^154^, and interfering with SCAR/WAVE activity impairs macropinocytosis ^155^. Of note, members of the Ras- and Rho-family are recruited/retained at the PM by electrostatic means: the negative surface charge of the inner leaflet attracts the polycationic C-terminus of the GTPases. Phosphoinositides contribute markedly to this effect by virtue of their polyvalency ^91^. It is conceivable that neutralization of this interaction by accumulation of submembranous H ^+^ accounts for the effects of amiloride, an inhibitor of Na ^+^/H ^+^ exchange that is commonly used to block macropinocytosis ^156^.

The mechanisms underlying closure of the macropinocytic cup and fission from the PM remain poorly understood but likely share some features with other endocytic pathways. Closure requires disassembly of submembranous actin, and this is effected, in part, by the hydrolysis of PtdIns(4,5)P _2_ catalyzed by PLC. PtdIns(3,4,5)P _3_ is required for the recruitment and activation of PLC ^137, 149^, specifically PLCγ1 ^157– 159^ and PLCβ ^160^. In addition, PtdIns(3,4,5)P _3_ can aid in scission directly through the recruitment of myosin proteins and indirectly through its hydrolysis products. In this regard, the disappearance of PtdIns(3,4,5)P _3_ during macropinocytosis coincides with the appearance of PtdIns(3,4)P _2_^132, 145, 161, 162^. In mammalian cells, SHIP2 is responsible for the dephosphorylation of PtdIns(3,4,5)P _3_ to PtdIns(3,4)P _2_^161^, and its depletion abrogates fluid-phase uptake of dextran ^163^, a reliable measure of macropinocytosis. The OCRL-like protein Dd5P4 performs the equivalent dephosphorylation reaction in *Dictyostelium* and is similarly required to support cup closure ^164^. Why this phosphoinositide conversion is required is not completely clear. A recent screen identified SNX9-family members (SNX9, SNX18, SNX33) as positive regulators of macropinocytosis ^165^. It is noteworthy that these are PX-BAR domain-containing proteins whose recruitment is triggered by PtdIns(3,4)P _2_ in other settings ^35^. It is unclear, however, whether BAR domains contribute to the shaping of membrane ruffles or promote fission in macropinocytosis, as they do in FEME and CME. It is also remarkable that not only is the production of PtdIns(3,4)P _2_ important for macropinocytosis but so too is its hydrolysis to PtdIns(3)P by INPP4B ^161^. What role PtdIns(3)P plays in the process is not yet understood.

## The internalization of pathogens

It is becoming increasingly evident that many pathogens harness or mimic intrinsic machinery of host cells for their benefit. By commandeering the hosts’ endocytic pathways, a variety of microorganisms have evolved means of gaining entry to cells and surviving within them. For example, numerous viruses and protozoa utilize macropinocytosis to mediate their cellular entry ^166, 167^. Other pathogens such as *Listeria monocytogenes* enter host cells by engaging surface receptors that can undergo CME and CIE by FEME ^14^. Here we highlight three selected pathogens that enter cells by diverse means and nestle into distinct host cell niches: *Salmonella enterica* drives its own cellular entry to reside in an endocytic compartment, *Legionella pneumophila* “hitches a ride” into alveolar macrophages by phagocytosis and generates a unique intracellular compartment, and enteropathogenic *Escherichia coli* (EPEC) manipulates the submembranous cytoskeleton to prevent its internalization, attaching firmly to the outer surface of host epithelial cells. While differing vastly in their survival strategies, these pathogens share a key aspect of their survival strategy: the subversion of the host’s phosphoinositide metabolism.

### *Salmonella enterica* 

*Salmonella enterica* spp. are a major worldwide cause of food-borne gastroenteritis (serovar Typhimurium) and Typhoid fever (serovar Typhi and Paratyphi) ^168– 171^. *Salmonella* gains entry into host cells and survives therein by virtue of effector proteins that are translocated into the host cytosol by type III secretion systems (T3SS) that are encoded in defined genomic pathogenicity islands ^172, 173^. *Salmonella* pathogenicity island-1 promotes the invasion of epithelial cells and the formation of *Salmonella*-containing vacuoles (SCV), while *Salmonella* pathogenicity island-2 promotes intracellular bacterial growth ^174^. Phosphoinositides play a key role in these processes.

**Figure 2.**
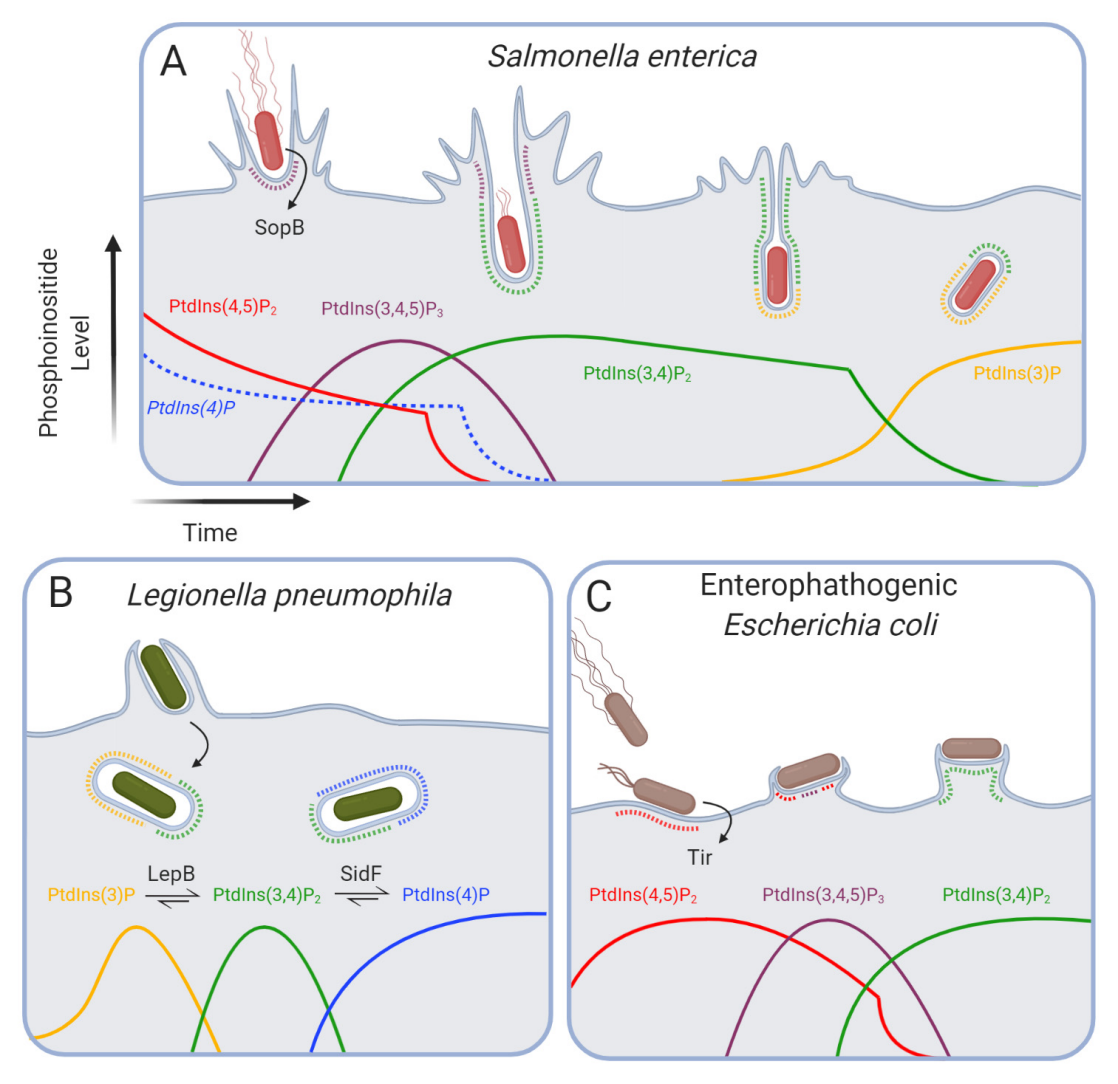
Bacterial effectors alter early endocytic traffic by subverting phosphoinositide metabolism. ( **A**) The *Salmonella enterica* effector SopB promotes efficient invasion of host cells by reducing PtdIns(4,5)P _2_ levels in invasion pockets while stimulating the production of PtdIns(3,4,5)P _3_, PtdIns(3,4)P _2_, and PtdIns(3)P. ( **B**) Following phagocytosis, *Legionella pneumophila* secretes multiple host effectors that modify phosphoinositides and disrupt early phagosome maturation. LepB functions as a phosphatidylinositide 4-kinase to generate PtdIns(3,4)P _2_ from PtdIns(3)P, and SidF is a 3-phosphatase that hydrolyzes PtdIns(3,4)P _2_ to produce PtdIns(4)P. ( **C**) Following contact with the host membrane, enteropathogenic *Escherichia coli* stimulates a transient increase in local PtdIns(4,5)P _2_ levels. The secreted bacterial effector Tir mediates the activation of host phosphatidylinositide 3-kinases to generate PtdIns(3,4,5)P _3_ and PtdIns(3,4)P _2_. PtdIns, phosphatidylinositol; Tir, translocated intimin receptor.

One effector secreted during invasion, SopB (also known as SigD), acts at least in part by co-opting the inositide metabolism of host cells. It is an important determinant of *Salmonella* virulence, contributing to inflammation and fluid secretion from infected ileum ^175, 176^. SopB shares homology with the mammalian phosphatases INPP4A/4B ^176^ and Synj ^177^. This predicted activity is borne out experimentally: *in vitro* SopB functions as a phosphatase of broad substrate specificity ^176, 177^. *In vivo*, however, only the 4-phosphatase activity of SopB has been demonstrated ( Figure 2A). Inside mammalian cells, it hydrolyzes plasmalemmal PtdIns(4,5)P _2_^178, 179^, generating PtdIns(5)P ^179^. SopB has also been suggested to possess 5-phosphatase activity ^177, 180^, but this requires further investigation. Regardless of the specific sites it can dephosphorylate, deletion of SopB results in the failure to clear PtdIns(4,5)P _2_ from invaginating regions of the membrane where invasion normally occurs, severely delaying scission of the SCV from the PM ^178, 181^. As a result, the invasion efficiency is markedly decreased ^180, 182^. The phosphoinositide changes brought about by SopB likely facilitate scission by reducing the rigidity of the cytoskeleton underlying the PM, in the process stimulating endocytosis ^178, 183^. Of note, *Shigella flexneri* ^184^—the causative agent of Shigellosis ^185^—secretes an effector known as IpgD that is structurally homologous to SopB and seemingly shares its hydrolytic activity towards phosphoinositides.

The manner whereby SopB (and presumably also IpgD) manipulates the host cell cytoskeleton to enable invasion is beginning to be understood. Through its phosphoinositide phosphatase activity, SopB can activate RhoG ^186^ and also acts as a guanine nucleotide-dissociation inhibitor towards Cdc42 ^187^. These effects act in concert with those triggered by separate effectors, like SopE and SopE2, that operate as Rho-family GTPase GEFs for Cdc42, Rac1, RhoA, and RhoG ^188– 190^, the antagonizing GAP SptP ^191, 192^, and SipA and SipC, which are actin-bundling proteins ^193– 195^. Together, these *Salmonella* effectors induce the formation of formin-mediated actin bundles, followed by Arp2/3-driven branched actin waves that jointly promote ruffle formation and closure, resulting in encapsulation of the bacterium ^194, 196– 199^.

Intriguingly, despite acting as a phosphatase, SopB mediates the formation of the 3-phosphorylated species PtdIns(3,4,5)P _3_ and PtdIns(3,4)P _2_ at invasion ruffles ^200, 201^ ( Figure 2A). These phosphoinositides recruit and activate AKT through its PH domain, thereby promoting host cell survival following infection ^202, 203^. In the same manner described for endogenous endocytosis, the 3-phosphorylated inositides produced by SopB support the recruitment of the PX-BAR domain-containing proteins SNX9 ^180^ and SNX18 ^204^, likely facilitating dynamin-mediated scission of the SCV from the PM.

The mechanism responsible for the formation of 3-phosphorylated species by SopB is not obvious; it is noteworthy, however, that it is insensitive to classical inhibitors of class I PI3Ks ^200, 201^. A possible target of the effector is PI3K-C2α, a class II PI3K resistant to conventional class I inhibitors ^34, 205^. In this regard, it is interesting that PI3K-C2α is co-opted by *Shigella flexneri* to promote its cell-to-cell spread ^206^ and that IpgD may be involved in the spreading process ^207, 208^. These observations raise the possibility that the homologous SopB may activate this enzyme during invasion. Finally, SopB contributes to the generation of PtdIns(3)P on the nascent SCV ^200^ by recruiting Rab5 and its effector, the class III PI3K Vps34.

### *Legionella pneumophila* 

*Legionella pneumophila*, the causative agent of Legionnaire’s disease, is another pathogen able to commandeer the phosphoinositide metabolism of its host cells. *Legionella* is internalized by alveolar macrophages following inhalation of aerosolized bacteria ^209– 211^. Once ensconced within the macrophage, *Legionella* utilizes a defective in organelle trafficking/intracellular multiplication (Dot/Icm) type IV secretion system to inject effectors across the phagosomal membrane—which becomes the *Legionella*-containing vacuole or LCV—to manipulate host cell pathways ^212, 213^. One such effector, LepB, alters the levels of cellular PtdIns(3)P and PtdIns(3,4)P _2_^70, 214^ ( Figure 2B). *In vitro* kinase assays using purified LepB found that LepB functions as a phosphatidylinositide 4-kinase, generating PtdIns(3,4)P _2_ from PtdIns(3)P, using ATP as a phosphate source ^214^.

PtdIns(3,4)P _2_ formed during *Legionella* infection is eliminated by another bacterial effector, the lipid phosphatase SidF, which hydrolyzes the D3 phosphate of PtdIns(3,4)P _2_ to produce PtdIns(4)P on the LCV ^215^ ( Figure 2B). The net result of these coordinated effector activities is to deplete PtdIns(3)P from the LCV by converting it first to PtdIns(3,4)P _2_, which is in turn hydrolyzed to PtdIns(4)P. By depleting PtdIns(3)P, *Legionella* appears to benefit from arresting maturation at an early stage. Moreover, the sustained production of PtdIns(4)P contributes to the maintenance on the LCV of effectors that specifically bind this inositide ^215– 217^ and enable fusion with secretory vesicles derived from the ER ^215^.

Through its ability to generate PtdIns(3,4)P _2_, LepB would be predicted to additionally activate AKT-dependent pro-survival host pathways ^55^. However, the dephosphorylation by SidF antagonizes this effect. Moreover, the supply of PtdIns(3)P to the LCV may be limited by VipD by blocking Rab5-dependent recruitment of Vps34 and/or through its phospholipase activity ^218, 219^. More detailed studies will be required to better establish the source and dynamics of these phosphoinositides on the LCV and their consequences on the effectors that determine bacterial virulence.

### Enteropathogenic *Escherichia coli*

In developing countries, EPEC present in contaminated food and water is a major cause of infant diarrhea and fatality ^220, 221^. Unlike the bacterial species discussed above, EPEC is predominantly an extracellular pathogen that, in fact, actively inhibits its own endocytosis. Instead, it creates a niche on the surface of the gastrointestinal epithelium by adhering tightly and replicating on actin-rich cell surface structures termed “pedestals”. EPEC utilize a T3SS to deliver effectors across the bacterial cell wall and host cell membrane, which promote adherence to epithelial cells lining the gastrointestinal tract, the loss of their microvilli (lesioning), and altered ion homeostasis, ultimately compromising barrier function ^222^. One such effector, translocated intimin receptor (Tir), integrates into the host PM and also couples to the bacterium by interacting with the intimin receptor on its outer membrane ( Figure 2C) ^223^. The Tir:intimin receptor complex is critical to dock EPEC onto the epithelial cell surface and in addition initiates signaling cascades. Like Fcγ phagocytic receptors, the cytosolic domain of Tir bears ITAM/ITIM-like motifs that can be phosphorylated on tyrosine residues by several host kinases ^224– 226^. Following its lateral clustering upon binding to intimin, Tir is phosphorylated and mediates the formation of the actin pedestal on which the adherent bacteria rest ^224, 227, 228^.

During infection, PtdIns(4,5)P _2_ transiently accumulates at the initial contact point between bacteria and the PM. The temporary increase occurs just after bacterial adherence and correlates with an accumulation of type I PIP5K and F-actin beneath the adhering bacteria ^229, 230^. The accumulation of PtdIns(4,5)P _2_ at the forming pedestal is largely dependent on a functional T3SS ^230^, although the effectors driving the recruitment of PIP5K remain unknown. Tir clustering, via the adaptor Nck, then recruits N-WASP, which activates the polymerization of branched actin by the Arp2/3 complex ^227, 228, 231^. It is worth noting that several other EPEC effectors play a part in modulating and polarizing the actin cytoskeleton: EspF co-opts SNX9 to further activate N-WASP ^232^, while EspH inactivates several RhoGEFs ^233^ and EspG interferes with activation of the WAVE complex ^234^. Regardless of the specific bacterial and cellular effectors engaged, the importance of PtdIns(4,5)P _2_ in the process is undeniable: the artificial enzymatic depletion of the inositide from the PM reduces bacterial adherence and pedestal formation ^230^.

Interestingly, Tir stimulates the production of PtdIns(3,4,5)P _3_ at least in part by binding and recruiting the p85 regulatory subunit to the membrane to activate class I PI3Ks ^230^. In accordance with this observation, Tir activates AKT pro-survival signaling ^230^; consequently, pharmacological inhibition of PI3Ks increases host cell death in response to EPEC ^235^, likely by inhibiting the production of the inositides necessary for AKT activation. It is likely that both the conversion of PtdIns(4,5)P _2_ to PtdIns(3,4,5)P _3_ and its hydrolysis by PLC—which is activated by Tir ^236^—contribute to the biphasic nature of PtdIns(4,5)P _2_ accumulation during pedestal formation ( Figure 2C).

More recently, the phosphoinositide phosphatase SHIP2 was identified as a host factor that is recruited to EPEC pedestals; the SH2 domain of SHIP2 associates with phosphorylated tyrosine residues on Tir. This interaction is functionally significant, since mutation of the tyrosine residues in the cytosolic domain of Tir or the depletion of SHIP2 led to the formation of disordered pedestals consisting of discrete actin-rich protrusions ^237^. How does SHIP2 regulate the organization of the pedestal? Rather than favoring sustained accumulation of PtdIns(3,4,5)P _3_, the phosphatase activity of SHIP2 generates a local platform rich in PtdIns(3,4)P _2_ in the pedestal ( Figure 2C). The latter inositide is sensed by the PH domain of lamellipodin, which is recruited to modulate F-actin polymerization in the pedestal ^67, 237^.

## Concluding remarks

The involvement of phosphoinositides in the generation of endocytic compartments and in directing their fate is now widely recognized, though not yet fully elucidated. The purpose of this review was to provide a bird’s-eye view of the current knowledge of the field. It is important to note that our survey of the literature was limited to the entry and early maturation stages. The role of inositides in late endosomes and lysosomes and in the equivalent stages of maturation of phagosomes and macropinosomes has only begun to be studied recently and should become the subject of a comprehensive and integrated view in the next few years as more information accumulates.

Lastly, it is important to note that the realization that inositides participate in membrane invagination, scission, and maturation was made possible primarily by the development and implementation of phosphoinositide-specific fluorescent probes that enabled real-time visualization of the individual lipid species with sufficient spatial and temporal resolution. In this regard, it is noteworthy that of the seven phosphoinositide species, suitable specific probes exist for only five of them ^238– 244^; to our knowledge, no satisfactory reagents are currently available to detect PtdIns5P or PtdIns(3,5)P _2_. Whether and how these lipids participate in membrane internalization and pathogen invasion remains to be studied as we await the development of suitable analytical tools.
